# Correlation between the Level of Inflammatory Cytokines and Prognosis in Children with IVIG-sensitive Kawasaki Disease and IVIG-resistant Kawasaki Disease

**DOI:** 10.12669/pjms.38.5.5408

**Published:** 2022

**Authors:** Hao Zhang, Hao-bin Song, De-xing Wang, Hong-yu Deng, Wen-long Sun

**Affiliations:** 1 Hao Zhang, Department of Cardiology, Baoding Children’s Hospital, Baoding 071000, China; Department of Cardiology, Baoding City Children Respiratory and Digestive Diseases Clinical Research key Laboratory, Baoding 071000, China; 2 Hao-bin Song, Department of Laboratory Medicine, Baoding Children’s Hospital, Baoding 071000, China; Department of Cardiology, Baoding City Children Respiratory and Digestive Diseases Clinical Research key Laboratory, Baoding 071000, China; 3 De-xing Wang, Baoding City Children Respiratory and Digestive Diseases Clinical Research key Laboratory, Baoding 071000, China; 4 Hong-yu Deng, Department of Laboratory Medicine, Affiliated Hospital of Hebei University, Baoding 071000, Hebei, China; 5 Wen-long Sun, Department of Laboratory Medicine, Baoding People’s Hospital, Baoding, Hebei 071000, China

**Keywords:** Children, Kawasaki disease, Coronary artery lesion, IVIG resistance

## Abstract

**Objectives:**

To investigate whether the levels of interleukin 1β (IL-1β), interferon γ (IFN-γ), tumor necrosis factor α (TNF-α) in children with Kawasaki disease (KD) are correlated with coronary artery lesion (CAL) and resistance to intravenous immunoglobulin (IVIG) treatment.

**Methods:**

A total of 216 children in line with KD diagnostic criteria were continuously included as subjects, and 50 healthy children at the same period were selected as the control group, and their levels of IL-1β, IFN-γ, and TNF-α were detected.

**Results:**

Subjects were subdivided according to the presence or absence of CAL: 42 cases (19.4%) of 216 children with KD developed CAL and were subdivided into the CAL group, while 174 (80.6%) of those who did not develop CAL were subdivided into the NCAL group. The levels of IL-1β, IFN-γ, and TNF-α in the CAL group and the NCAL group were higher than those in the control group (P<0.05), and the levels of those in the CAL group were higher than those in the NCAL group (P<0.05). Subjects were subdivided according to the effect of IVIG treatment: 194 cases (89.8%) of 216 children with KD had a good control of inflammation after the initial IVIG treatment, and were considered to have IVIG-sensitive KD and divided into the IVIG-sensitive group; 22 cases (10.2%) could not get good control of inflammation after the initial IVIG treatment, and were considered to have IVIG-resistant KD and divided into the IVIG-resistant group. The levels of IL-1β, IFN-γ, and TNF-α in the IVIG-sensitive group and the IVIG-resistant group were higher than those in the control group; The levels of IL-1β, IFN-γ, and TNF-α in the IVIG-resistant group were higher than those in the IVIG-sensitive group (P<0.05), while the fever time of the IVIG-sensitive group was lower than that of the IVIG-resistant group (P<0.05).

**Conclusion:**

Children with KD may experience changes in IL-1β, IFN-γ, and TNF-α levels in the acute phase. Such a significant increase in levels may be a risk factor for CAL and resistance to IVIG treatment in children with KD, while the prolonged fever time is a risk factor for resistance to IVIG treatment in children with KD.

## INTRODUCTION

Kawasaki disease (KD), an acute febrile disease with unknown etiology that occurs mostly in children, may give rise to systemic vasculitis. Some children with KD may even develop coronary artery lesion (CAL) or even coronary aneurysm.^1^ Currently, IVIG and aspirin are the preferred for the treatment of KD. However, some children with KD have poor outcomes after initial IVIG treatment. Part of children with KD will still develop CAL after timely treatment. Moreover, approximately 10% to 20% of patients with KD are considered to be suffering from IVIG-resistant KD when their body temperature fails to drop to normal or fever occurs again in a short time after IVIG treatment.^2^ Such children with poor curative effect are the focus of diagnosis and treatment for KD at present. It is considered in current studies that inflammatory cytokines exert an important part in the course of KD, which has a close bearing on the appearance of CAL as well as IVIG resistance in children with KD.^2^,^3^ In this study, the levels of interleukin 1β (IL-1β), interferon γ (IFN-γ) and tumor necrosis factor α (TNF-α) in children diagnosed with KD in our hospital were detected, so as to find out whether the levels of inflammatory cytokines are correlated with the sensitivity of children with KD to IVIG treatment and CAL.

## METHODS

In this study, 216 children diagnosed with KD in our hospital from June 2017 to June 2020 were selected as subjects, including 121 males and 95 females, aged from 1 to 8 years, with an average age of 3.8±1.7 age. Fifty healthy children who underwent physical examination in our hospital at the same time were selected as the control group, including 28 males and 22 females, aged from 1 to 8 years, with an average age of 3.8±1.8 years. No statistical significance can be seen in the comparison of gender and age between the two groups (P>0.05).

### Ethical Approval:

The study was approved by the Institutional Ethics Committee of Laboratory of Baoding Children’s Hospital.( September 17, 2021)

KD diagnostic criteria issued by the American Heart Association in 2017[4]: Fever ≥ 5d, with at least 4 of 5 clinical manifestations, and other diseases need to be excluded: 1. Erythema and edema of the hands and feet in the acute and/or subacute phases, and desquamation of the ends of fingers and toes within 2-3 weeks of fever; 2. Erythema rash; 3. Bilateral bulbar conjunctival non-exudative conjunctivitis; 4. Changes in the lips and mouth include: (1) Erythema, dryness, peeling, cracking and bleeding on the lips; (2) “Strawberry Tongue”; (3) Diffuse erythema; 5. Swollen lymph nodes in the neck are usually unilateral and ≥1.5 cm in diameter.

### CAL judgment criteria^5^:

The occurrence of CAL is defined according to Z score: 1. No CAL: < 2; 2. Coronary artery dilation: 2 - < 2.5; 3. Aneurysm: ≥ 2.5.

### Judgment criteria for IVIG treatment of resistant KD^6^:

Persistent fever within 36h after initial IVIG treatment, or recurrent fever after the body temperature became normal (body temperature ≥ 38°C), without secondary infection.

### Exclusion criteria:

- Patients with severe organic diseases such as heart, liver and kidney diseases or diseases such as genetic metabolic diseases, immune diseases, allergic diseases and tumors;
- Patients who have taken drugs such as immunomodulators and hormones within six months;
- Patients with infectious diseases;
- Patients who cannot cooperate to complete the study.

### Specimen Collection:

2 mL of venous blood was collected on an empty stomach from children with KD within 5-7 days of onset and before treatment with IVIG and aspirin.

### Detection method of IL-1β, IFN-γ, TNF-α:

In this study, the detection was carried out by double-sandwich microsphere capture method, and the reagents used were all purchased from Beijing QuantoBio Biotechnology Co., Ltd. The instrument used in the experiment was MindyayBriCyte E6 flow detector. The detection of this study was carried out by Baoding Key Laboratory of Clinical Research on Children’s Respiratory and Digestive Diseases and Baoding Precision Diagnosis and Treatment Laboratory of Infectious Diseases of Children.

### Statistical Analysis:

All the data in this study were processed by SPSS 22.0 statistical software. Measurement data were expressed as X̄±S, and ANOVA was used to compare the selection between groups. P<0.05 indicates a statistically significant difference.

## RESULTS

Grouping according to the presence or absence of CAL: 42 cases (19.4%) of 216 children with KD developed CAL and were subdivided into the CAL group, including 24 males and 18 females with an average age of 3.9±1.9 years; 174 (80.6%) of those who did not develop CAL were subdivided into the NCAL group, including 97 males and 77 females, with an average age of 3.8±1.7 years.

Grouping according to the effect of IVIG treatment: 194 cases (89.8%) of 216 children with KD had a good control of inflammation after the initial IVIG treatment, and were considered to have IVIG-sensitive KD and divided into the IVIG-sensitive group, including 108 males and 86 females, with an average age of 3.8±1.7 years; 22 cases (10.2%) could not get good control of inflammation after the initial IVIG treatment, and were considered to have IVIG-resistant KD and divided into the IVIG-resistant group, including 13 males and 9 females, with an average age of 3.8±1.9 years.

The levels of IL-1β, IFN-γ and TNF-α in the CAL group and the NCAL group were higher than those in the control group, with a statistically significant difference (P<0.05). The levels of IL-1β, IFN-γ and TNF-α in the CAL group were higher than those in the NCAL group, with a statistically significant difference (T=5.42, T=4.74, T=7.87; P<0.05), Table-I.

**Table I:** Comparison of IL-1β, IFN-γ and TNF-α levels between the CAL group, the NCAL group and the control group.

Group	Number of cases	IL-1β (pg/mL)	IFN-γ (pg/mL)	TNF-α (pg/mL)
CAL group	42	23.6±11.6	25.4±14.7	20.0±7.9
NCAL group	174	13.6±5.8	14.5±5.9	10.2±3.7
Control group	50	2.4±1.2	2.9±1.4	2.9±1.6
F		120.31	101.98	174.65
P		0.00	0.00	0.00

The levels of IL-1β, IFN-γ and TNF-α in the IVIG-sensitive group and the IVIG-resistant group were higher than those in the control group, with a statistically significant difference (P<0.05). The levels of IL-1β, IFN-γ and TNF-α in the IVIG-resistant group were higher than those in the IVIG-sensitive group, with a statistically significant difference (T=2.58, T=2.15, T=2.36; P<0.05), Table-II.

**Table II:** Comparison of IL-1β, IFN-γ and TNF-α levels between the IVIG-sensitive group, the IVIG-resistant group and the control group.

Group	Number of cases	IL-1β (pg/mL)	IFN-γ (pg/mL)	TNF-α (pg/mL)
IVIG-sensitive group	194	15.0±7.4	15.9±8.3	11.8±5.9
IVIG-resistant group	22	21.4±11.5	22.9±15.1	15.0±7.7
Control group	50	2.4±1.2	2.9±1.4	2.9±1.6
F		76.63	62.71	58.68
P		0.00	0.00	0.00

The fever time of the IVIG-sensitive group was lower than that of the IVIG-resistant group, with a statistically significant difference (P<0.05), Table-III.

**Table III:** Comparison of fever time between the IVIG-sensitive group and the IVIG-resistant group.

Group	Fever time
IVIG-sensitive group	7.4±1.1
IVIG-resistant group	10.3±1.9
t	7.11
p	0.00

## DISCUSSION

In the acute stage of KD, vascular endothelial injury will occur, and the combination of anti-endothelial autoantibodies and endothelial cells will lead to the activation or injury of endothelial cells and the release of pro-inflammatory cytokines, resulting in coronary artery lesions.^7^ A single intravenous injection of 2 g/kg of immunoglobulin and aspirin can reduce the incidence of coronary aneurysms from 25% to 5%. However, 10-20% of patients do not respond to this treatment and suffer an increased risk of cardiac complications and death.^8^

Inflammatory cytokines such as IL-1β, IFN-γ, and TNF-α are involved in the pathogenesis of KD, and may affect the prognosis of KD in various aspects. For patients with acute KD, their T, B lymphocytes and monocyte/macrophage system will be activated, which will lead to the large production of immunoglobulins and inflammatory cytokines in the body. Inflammatory cytokines such as IL-1 and TNF-α can also promote the aggregation and activation of inflammatory cells, and promote the release of inflammatory mediators, resulting in inflammatory symptoms such as fever. Such an increase in these cytokines may be closely related to IVIG resistance.^9^-^11^ Matrix metalloproteinase (MMP) is involved in the extracellular remodeling of KD and is associated with coronary artery lesion in patients with KD. IL-1β, IL-6 and TNF-α stimulate the expression of MMP-9, while IFN-γ inhibits the expression of MMP-9.^12^ IL-1β is of vital inportance in the pathophysiological mechanism of cardiovascular disease and KD.^13^,^14^ IL-1β may trigger vasodilation induced by local pro-inflammatory environment and attract monocytes and neutrophils to the site causing tissue damage and stress, which is closely related to KD inflammation and aneurysm formation.^15^ It is considered in current studies that the levels of IL-1β, IFN-γ, and TNF-α in KD children with CAL will be significantly higher than those of children with KD without CAL.^16^-^18^ As shown in this study, the levels of IL-1β, IFN-γ, and TNF-α in children with KD (with or without CAL) were significantly higher than those in healthy children, and the levels of IL-1β, IFN-γ and TNF-α in KD children with CAL were significantly higher than those in KD children without CAL, which was consistent with previous reports. It is suggested that IL-1β, IFN-γ and TNF-α were not only involved in the pathological process of KD, but also the increase of their levels was closely related to CAL in children with KD.

It has been reported that the levels of interferon γ (IFN-γ) and tumor necrosis factor α (TNF-α) in children with IVIG-resistant KD are significantly higher than those in children with IVIG-sensitive KD.^2^,^18^ The genetic polymorphism of IL-1β is associated with resistance to IVIG in children with KD.^19^ Changes in IL-1β level exerts a crucial role in the resistance to IVIG treatment in children with KD.^20^ In this study, the fever time, IL-1β, IFN-γ and TNF-α levels in the IVIG-resistant group were higher than those in the IVIG-sensitive group, which was consistent with previous reports. It is suggested that children with KD have higher levels of inflammatory cytokines early in the course of the disease, so it is necessary to be alert to the possibility of resistance to IVIG treatment, closely monitor the temperature and inflammation indicators of the children, and timely communicate with their families to avoid delay of the disease.

### Limitation of the study:

Few cases were included and no multi-center study was conducted.

## CONCLUSION

Children with KD may experience changes in IL-1β, IFN-γ, and TNF-α levels in the acute phase. Such a significant increase in levels may be a risk factor for CAL and resistance to IVIG treatment in children with KD, while the prolonged fever time is a risk factor for resistance to IVIG treatment in children with KD.

### Authors’ Contributions:

**HZ** & **HBS:** Designed this study and prepared this manuscript.

**DXW, HYD:** Collected and analyzed clinical data.

**WLS:** Significantly revised this manuscript.

## References

[1] Noval Rivas M, Arditi M (2020). Kawasaki disease:pathophysiology and insights from mouse models. Nat Rev Rheumatol.

[2] Wang Y, Qian SY, Yuan Y, Wang Q, Gao L, Chen X (2020). Do cytokines correlate with refractory Kawasaki disease in children?. Clin Chim Acta.

[3] Feng S, Su Y, Luo L, Jing F, Yi Q (2018). Serum levels of C1q/tumor necrosis factor-related protein-1 in children with Kawasaki disease. Pediatr Res.

[4] McCrindle BW, Rowley AH, Newburger JW, Burns JC, Bolger AF, Gewitz M (2017). Diagnosis, Treatment, and Long-Term Management of Kawasaki Disease:A Scientific Statement for Health Professionals From the American Heart Association [published correction appears in Circulation. 2019 Jul 30;140(5):e181-e184]. Circulation.

[5] Dimitriades VR, Brown AG, Gedalia A (2014). Kawasaki disease:Pathophysiology, clinical manifestations, and management. Curr Rheumatol Rep.

[6] Fu LY, Qiu X, Deng QL, Huang P, Pi L, Xu Y (2019). The IL-1B Gene Polymorphisms rs16944 and rs1143627 Contribute to an Increased Risk of Coronary Artery Lesions in Southern Chinese Children with Kawasaki Disease. J Immunol Res.

[7] Sakurai Y (2019). Autoimmune Aspects of Kawasaki Disease. J Investig Allergol Clin Immunol.

[8] Dusser P, Kone-Paut I (2017). IL-1 Inhibition May Have an Important Role in Treating Refractory Kawasaki Disease. Front Pharmacol.

[9] Sakata K, Hamaoka K, Ozawa S, Niboshi A, Yahata T, Fujii M (2010). Matrix metalloproteinase-9 in vascular lesions and endothelial regulation in Kawasaki disease. Circ J.

[10] Franco A, Shimizu C, Tremoulet AH, Burns JC (2010). Memory T-cells and characterization of peripheral T-cell clones in acute Kawasaki disease. Autoimmunity.

[11] Wu Y, Liu FF, Xu Y, Wang JJ, Samadli S, Wu YF (2019). Interleukin-6 is prone to be a candidate biomarker for predicting incomplete and IVIG nonresponsive Kawasaki disease rather than coronary artery aneurysm. Clin Exp Med.

[12] Sakata K, Hamaoka K, Ozawa S, Niboshi A, Yahata T, Fujii M (2010). Matrix metalloproteinase-9 in vascular lesions and endothelial regulation in Kawasaki disease. Circ J.

[13] Heydari FS, Zare S, Roohbakhsh A (2020). Inhibition of Interleukin-1 in the Treatment of Selected Cardiovascular Complications [published online ahead of print, 2020 Jul 16]. Curr Clin Pharmacol.

[14] Porritt RA, Markman JL, Maruyama D, Kocaturk B, Chen S, Lehman TJA (2020). Interleukin-1 Beta-Mediated Sex Differences in Kawasaki Disease Vasculitis Development and Response to Treatment. Arterioscler Thromb Vasc Biol.

[15] Dusser P, Kone-Paut I (2017). IL-1 Inhibition May Have an Important Role in Treating Refractory Kawasaki Disease. Front Pharmacol.

[16] Guo C, Tan C, Xia X, Yuan Y, Zhao M, Yuan Z (2020). Tumour necrosis factor-αand myoglobin associated with the recovery time of coronary artery lesions in Kawasaki disease patients. J Paediatr Child Health.

[17] Feng S, Su Y, Luo L, Jing F, Yi Q (2018). Serum levels of C1q/tumor necrosis factor-related protein-1 in children with Kawasaki disease. Pediatr Res.

[18] Wang Y, Wang W, Gong F, Fu S, Zhang Q, Hu J (2013). Evaluation of intravenous immunoglobulin resistance and coronary artery lesions in relation to Th1/Th2 cytokine profiles in patients with Kawasaki disease. Arthritis Rheum.

[19] Weng KP, Hsieh KS, Ho TY, Huang SH, Lai CR, Chiu YT (2010). IL-1B polymorphism in association with initial intravenous immunoglobulin treatment failure in Taiwanese children with Kawasaki disease. Circ J.

[20] Tremoulet AH, Jain S, Kim S, Newburger J, Arditi M, Franco A (2016). Rationale and study design for a phase I/IIa trial of anakinra in children with Kawasaki disease and early coronary artery abnormalities (the ANAKID trial). Contemp Clin Trials.

